# The Evolutionary Development of the Brain As It Pertains to Neurosurgery

**DOI:** 10.7759/cureus.6748

**Published:** 2020-01-23

**Authors:** Jaafar Basma, Natalie Guley, L. Madison Michael II, Kenan Arnautovic, Frederick Boop, Jeff Sorenson

**Affiliations:** 1 Neurological Surgery, University of Tennessee Health Science Center, Memphis, USA; 2 Neurological Surgery, University of Arkansas for Medical Sciences, Little Rock, USA; 3 Neurological Surgery, Semmes-Murphey Clinic, Memphis, USA

**Keywords:** anatomy, comparative, brain neoplasms, cerebrum, phylogeny, limbic system, neuroanatomy, neurosurgery

## Abstract

Background

Neuroanatomists have long been fascinated by the complex topographic organization of the cerebrum. We examined historical and modern phylogenetic theories pertaining to microneurosurgical anatomy and intrinsic brain tumor development.

Methods

Literature and history related to the study of anatomy, evolution, and tumor predilection of the limbic and paralimbic regions were reviewed. We used vertebrate histological cross-sections, photographs from Albert Rhoton Jr.’s dissections, and original drawings to demonstrate the utility of evolutionary temporal causality in understanding anatomy.

Results

Phylogenetic neuroanatomy progressed from the substantial works of Alcmaeon, Herophilus, Galen, Vesalius, von Baer, Darwin, Felsenstein, Klingler, MacLean, and many others. We identified two major modern evolutionary theories: “triune brain” and topological phylogenetics. While the concept of “triune brain” is speculative and highly debated, it remains the most popular in the current neurosurgical literature. Phylogenetics inspired by mathematical topology utilizes computational, statistical, and embryological data to analyze the temporal transformations leading to three-dimensional topographic anatomy. These transformations have shaped well-defined surgical planes, which can be exploited by the neurosurgeon to access deep cerebral targets. The microsurgical anatomy of the cerebrum and the limbic system is redescribed by incorporating the dimension of temporal causality. Yasargil’s anatomical classification of glial tumors can be revisited in light of modern phylogenetic cortical categorization.

Conclusion

Historical and modern topological phylogenetic notions provide a deeper understanding of neurosurgical anatomy and approaches to the limbic and paralimbic regions. However, many questions remain unanswered and further research is needed to elucidate the anatomical pathology of intrinsic brain tumors.

## Introduction

Anatomists have long marveled at the intricate three-dimensional anatomy of the brain. Cerebral structures are woven together through elaborate spatial configurations, segregating along predictable fissures, sulci, and arachnoidal and ventricular spaces. From the early works of Alcmaeon, Herophilus, Galen, Vesalius, and others, to the currently employed state-of-the-art visual three-dimensional and digital technology, descriptive neuroanatomy has evolved in an attempt to further understand the brain’s perplexing architecture [1]. Still, unraveling the causal elements driving cerebral design can open the door to a deeper and more logical appreciation of the anatomy. While such an analytical study is appealing from a purely theoretical, historical, and epistemological perspectives, it also has significant applications to the clinical practice of neurosurgery [2].

Causal inferences are indispensable to neuroanatomy as they are in the medical fields of physiology, pharmacology, and pathology. According to philosopher David Hume, temporal contiguity is a necessary condition to establish a causal effect. Similarly, the study of phylogenetics and the speculated evolutionary development of the human brain incorporate the dimension of time into the description of anatomy [3].

In explaining why cerebral structures follow their observed morphology, these disciplines hold the keys to a more insightful appreciation of the normal topographic anatomy, surgical planes to deep cerebral structures, and even the development of several neurosurgical pathologies. Although this concept of so-called “four-dimensional” anatomy constitutes the foundation of modern phylogenetics [3,4], its application to neurosurgery has not been well reviewed in the literature. Furthermore, the evolutionary notions often discussed in neurosurgical descriptions can be considered outdated and inaccurate in light of modern phylogenetic theories.

In this paper, we review hypothesized topological transformations that explain specific topographic relationships of the brain. We present comparative anatomical brain sections of vertebrate species and human microsurgical dissections from Dr. Albert Rhoton, Jr.’s collection, in an attempt to describe a “four-dimensional” anatomy of the brain.

## Materials and methods

A thorough review of both historical and modern neuroanatomical literature was performed, focusing on evolution as it relates to cerebral microsurgical anatomy. Photographs from Professor Rhoton's cadaveric dissections and vertebrate histological cross-sections from the anatomy laboratory of the University of Tennessee Health Science Center (UTHSC) were used to assist the reader in assembling the presumed causal events leading to the development of human cerebral anatomy. Our ultimate intent was to incorporate the evolutionary narrative in explaining the topographic relationships of Rhoton’s microsurgical dissections.

Anatomical sectors of vertebrate brains

Animal sections were obtained from multiple previous and ongoing research projects at the anatomy department of the UTHSC. Animals were treated according to animal welfare guidelines of the National Institutes of Health, the Society for Neuroscience (SFN), and the U.S. Animal Welfare Act. Animal studies were approved by the Institutional Animal Care and Use Committee, UTHSC, Memphis (IACUC ID: 16-110) and USAMRMC Animal Care and Use Review Office (Protocol No. VR150072, 12/05/2016), following the guidelines of DOD Instruction 3216.01, “Use of Animals in DOD Programs.”

Whole Body Perfusion

All animals and non-human primates were deeply anesthetized (Avertin; 0.2 mL/g body weight), the chest was opened, and 0.1mL of heparinized saline (800 USP. units/mL) was injected into the left ventricle of the heart. They were then perfused transcardially with 0.9% NaCl in 0.1 M sodium phosphate buffer at pH 7.4 phosphate buffer (PB), followed by 4% paraformaldehyde, 0.1 M lysine-0.1 M sodium periodate in 0.1 M PB at pH 7.4 periodate-lysine-paraformaldehyde fixative (PLP). After perfusion, brains were carefully dissected and placed in 2% PLP to post-fix for a set period of time, which varied by species and brain size. For long-term storage, brains were moved into a 20% sucrose/10% glycerol solution and were stored at 4 °C.

Sectioning and Staining

Fixed brains were first frozen with dry ice and then sectioned on a sliding microtome in the transverse plane at 35 µm. Free-floating sections were stained with cresyl violet according to established laboratory protocol and mounted onto glass microscope slides. Finally, slides were dehydrated and cover-slipped with mounting medium.

Image Capture

Coronal sections at the level of the diencephalon were chosen for each species and high-resolution images were captured of the histology slides using a flatbed scanner (Epson Perfection V500, Epson America, Long Beach, CA).

## Results

Historical background

From Topographic to Comparative Anatomy

Alcmaeon of Croton, a pre-Socratic philosopher and physician, is credited with being the father of scientific topographic anatomy. While the prior anatomical treaties of Egyptian papyri were ritually focused, Alcmaeon adopted an observational methodology. Herophilus of Chalcedon and Galen of Pergamon advanced anatomical knowledge significantly in antiquity by utilizing systemic dissections of animals and human bodies. Their anatomical descriptions, however, were not thoroughly reviewed until the Renaissance era, primarily by Andreas Vesalius. Vesalius discovered many errors in ancient anatomical descriptions from observations he made from his own cadaveric dissections. Many of Galen’s mistakes were attributed to his reliance on animal studies, such as the oxen [1]. The noted similarities and differences in the anatomy of different species eventually lead to the establishment of the field of comparative anatomy. Karl Ernst von Baer listed the principles of homology, analogy, and homoplasty to classify species based on similar characteristics of their anatomical structures.

From the study of different species’ homology, Charles Darwin elaborated on his famous theory of evolution and natural selection [5,6]. Darwin proposed that these changes were not due solely to random chance but were driven by how well those changes allowed an organism to adapt to their natural environment (i.e., natural selection) [6]. The modern synthesis and the concept of genetic drift brought about a harmony between Darwinian evolution and Mendelian population genetics [7,8]. In more recent years, the role of epigenetics has drastically shifted our understanding of how organisms can change in real-time in response to alterations in the environment [9].

Early Development

Studies of the amphioxus, a small marine invertebrate species whose central nervous system is formed entirely of a spinal cord, suggest an early separation between the dorsal sensory and ventral motor regions by a limiting sulcus. The evolution and usefulness of decussation in the human brain have been a vexing problem for scientists [10]. The widely accepted explanation is that primitive vertebrates had to flex their body on the contralateral side to avert noxious stimuli, leading to a crossover between sensory and motor circuits. The brainstem then developed as a hyper-specialized spinal cord to accommodate functionality for special senses and primitive coordination of movements. Sensations related to the immediate environment (i.e., tactile, taste) were an early addition and are localized in the hindbrain. Distant sensations (i.e., vision, olfaction) emerged later and are hence located in the midbrain and forebrain. Hearing branched later as an adaptation of the vestibular system and vibratory perception [3].

Associative and correlative functions began to unfold in the midbrain (e.g., optic tectum). This led to the emergence of higher diencephalic centers. The thalamus anatomically constitutes a rostral continuation of the midbrain. The third ventricle is the most rostral structure of the original neural tube and is bordered superiorly by the lamina terminalis (hence its name). Basic vital functions are controlled by the hypothalamic nuclei on the floor of the ventricle. From the rostral end of the neural tube, the paired lateral outgrowths of the forebrain evolved to control behavior related to olfaction and active food-seeking. The forebrain became separated from the diencephalon by the lamina terminalis and the velum interpositum. Forebrain structures thus emerged rostrally and then expanded caudally, forming progressive concentric rings (i.e., limbic and paralimbic regions) around the primitive “salamander brain” (i.e., spinal cord, brainstem, thalamus) [3].

Triune Brain

In this “ladder” model proposed by Paul MacLean, advanced layers of the brain that were unique to certain species emerged above and supplemented the older lower structures. A paleo-mammalian complex (limbic and paralimbic system) conferring motivation, emotion, and memory developed above a reptilian complex (basal ganglia and olfactory cortex) associated with instinctual behavior and aggression. The neo-mammalian complex (neocortex) emerged most recently and its expansion displaced the older structures downwards and inwards. The neocortex added the abilities of language, planning, abstract thinking, and consciousness (Table 1) [11].

 

**Table 1:** Correlation between old, traditional, and new phylogenetic nomenclatures PHG: parahippocampal gyrus

New anatomical terminology	Old anatomical terminology	Cortical cytoarchitecture	Phylogenetic terminology	Triune phylogenetic terminology	Anatomical structures of mammals
Isocortex	Neocortex	Six layers	Dorsal pallium	Neomamallian complex	Frontal, temporal, parietal, occipital lobes
Mesocortex	Mesocortex	Transitional	Dorsal pallium	–	PHG, cingulate, orbitofrontal, anterior Insular, entorhinal
Allocortex	Paleocortex	Three layers	Lateral pallium	Paleomammalian complex	Piriform cortex
–	Archicortex	Three layers	Medial pallium	Paleomammalian complex	Hippocampus, dentate gyrus
Subcortical	Subcortical	–	Subpallium	Reptilian complex	Septum, striatum (caudate, putamen, accumbens), amygdala

**Figure 1:**
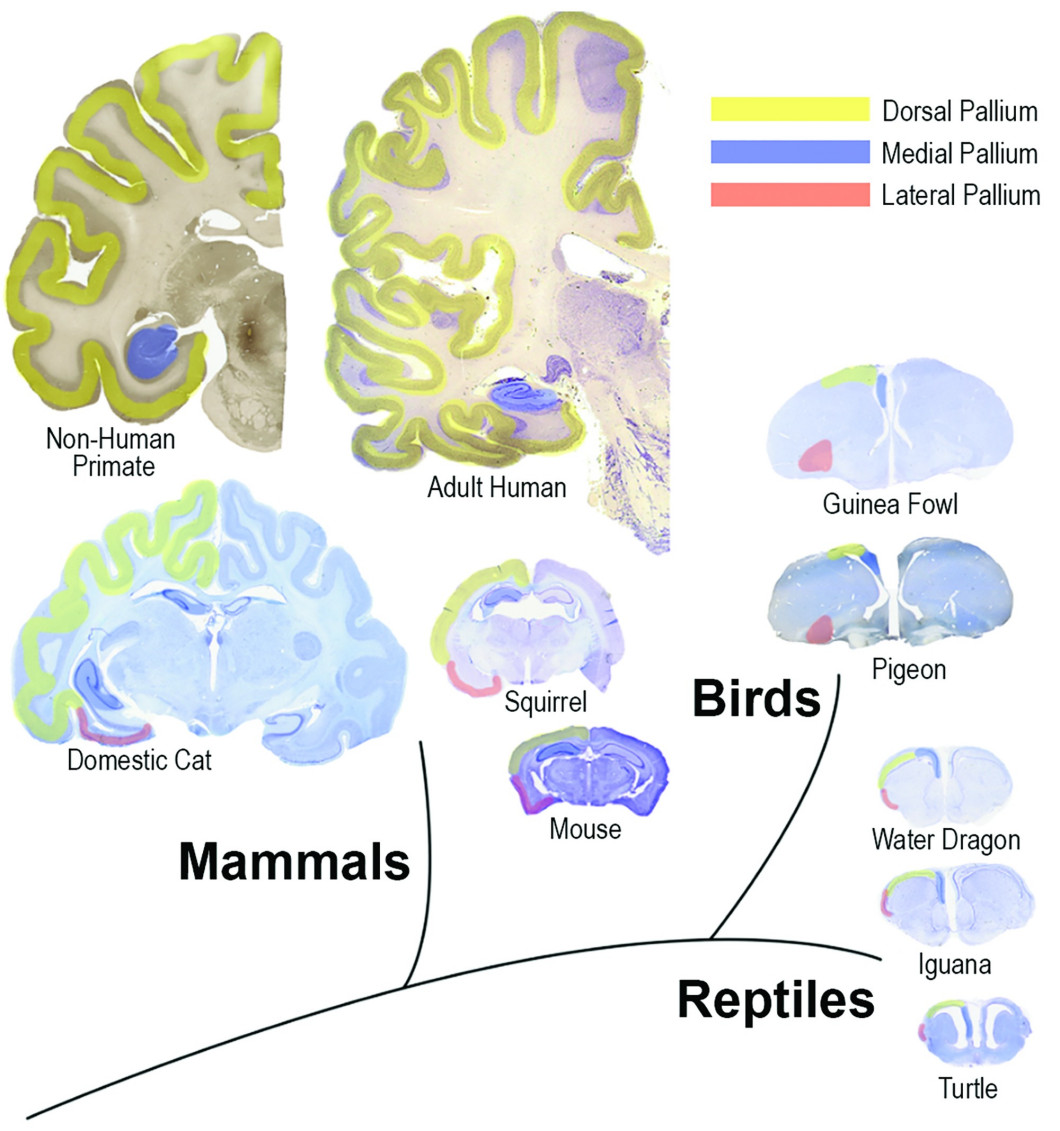
Comparative anatomical sections illustrating brains of adult human, non-human primate, domestic cat, squirrel, mouse, guinea fowl, pigeon, water dragon, iguana, and turtle Notice the rostral position of the medial pallium (precursor of hippocampus, colored in blue) at the roof of the ventricle in the squirrel and mouse, compared with its caudal position in humans at the floor of the temporal horn. The dorsal pallium (origin of neocortex) develops inward in reptiles to form the dorsal periventricular ridge (turtle), while it migrates to the surface away from the ventricles in mammals. Basal ganglia form a major part of the bird’s brain. The lateral pallium (olfactory regions, in red) occupies a relatively small area in the human brain Image credit for human brain cross-section: Allen Institute, Allen Human Brain Atlas, http://www.human.brain-map.org

Given the popularity of this model, the most commonly cited phylogenetic notions in the neurosurgical literature still derive from it [2,12]. But its inaccuracies were demonstrated by identifying basal ganglia structures in species primitive to reptiles, and limbic structures in non-mammals. Non-mammals were even shown to have periventricular structures homologous with the mammalian neocortex, called regions of the dorsal pallium (Figure 1) [13].

Topological Phylogenetics

In recent years, comparative anatomy has been complemented with computational genomics and mathematical topology [14]. Topology is the field of mathematics that contemplates the properties of space and objects in conditions of continuous deformations, such as the uninterrupted transformation of a primitive neural tube into a human brain.

In the “branching model” of evolution, differentiation occurs through separation and parcellation of ancestral structures [15]. Newer regions of the brain did not emerge spontaneously but rather branched out from an antecedent area without violating the topology (i.e., spatial continuity) of the original design. This concept of topological preservation was demonstrated in embryology. Rakic described the glial processes, which guide the migration of neurons from the periventricular zone to the cortical surface [16].

Topological transformations

General Organization

The dorsal wall of the hemisphere or pallium (Latin, meaning “covering”) has a medial part (eventually forming the hippocampus), a dorsal region (precursor of isocortex or neocortex), and a lateral region (piriform/olfactory lobe). The ventral wall of the hemisphere, or subpallium, develops into the septum medially, and the striatum and amygdala laterally (Figure 1) [4,17].

The amphibian brain has the closest design to the primitive vertebrate prototype. Its hemisphere is capped by the large olfactory bulb. Its pallium is still peri-ventricular and does not migrate to the surface. The reptilian brain diverged from the mammalian brain, but its medial pallium is also divided into a lateral portion (hippocampal) and a medial part (dentate gyrus). Its dorsal pallium forms the dorsal ventricular ridge, which is believed to be homologous with the mammalian isocortex (i.e., neocortex). However, it did not migrate outward to the surface and instead grew inward [17].

It is noted that the mammalian cortex differentiated early into three subtypes: allocortex, mesocortex, and isocortex. This nomenclature has been adopted in modern phylogenetics instead of the older division of paleocortex, archicortex, and neocortex (Table 1). Although the allocortex (i.e., the piriform and hippocampus) is close to the reptilian pallium in its cytoarchitecture, its topological transformation has significantly changed its topographic structure (interlocking “Cs” of dentate gyrus and cornu ammonis in mammals) [4].

Expansion of the Isocortex and Resultant Topological Rotations

**Figure 2:**
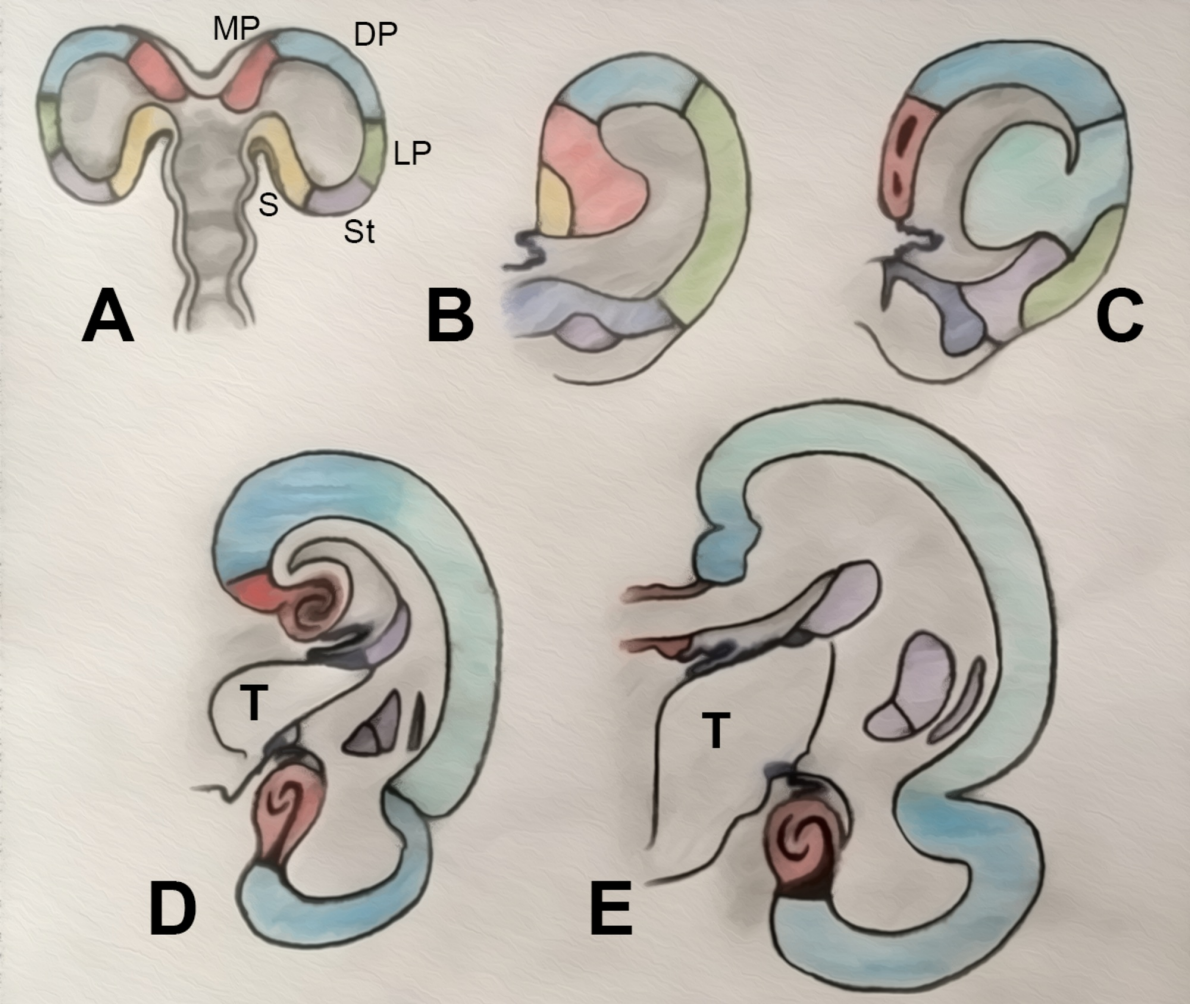
Illustration of the topological transformation of a prototypical vertebrate vesicle Topological transformation of a prototypical vertebrate vesicle (A) to amphibians (B), reptiles (C), acallosal mammals (D), and callosal mammals (E). The prototypical design of the brain contains three separate “pallia” – medial, dorsal, and lateral – that are present in their named positions in reptiles but have evolved in mammalian vertebrates to become the hippocampus, isocortex/neocortex, and the piriform cortex, respectively. The dorsal pallium forms the periventricular ridge in reptiles (C) and migrates to the surface to become the isocortex and mesocortex in mammals (D, E). The floor of the vesicle is formed by the striatum and the septum, thus separating the pallium from the diencephalic structures. Note that the medial wall of the hemisphere close to the foramen is relatively underdeveloped, eventually constituting the choroid fissure. Driven by the superficial growth of the cortex, the internal capsule and the thalamus expand the mesocortex, caudate and medial pallium from inside, leading to the formation of a “limbic ring.” (Adapted from Gloor) [4]. DP: dorsal pallium (dark blue: mesocortex; light blue: isocortex); LP: lateral pallium (green); MP: medial pallium (red); S: septum (yellow); St: striatum (purple); T: thalamus

**Figure 3:**
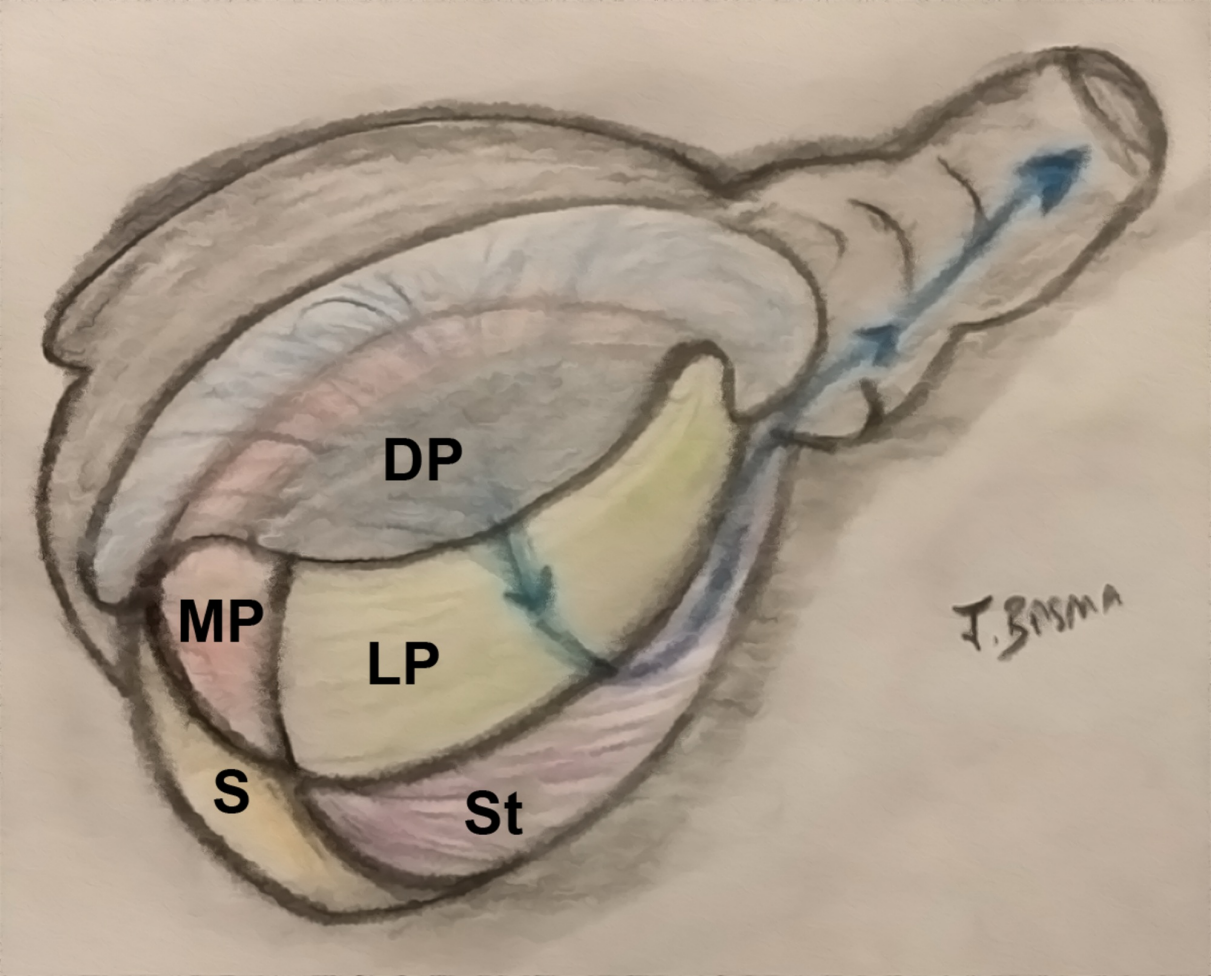
Illustration of the precursor brain regions in the Three dimensions Note the continuity between the medial and lateral pallia at the pole of the hemisphere. The dorsal pallium migrates superficially and expands, while keeping its connection with the thalamus and the brainstem. DP: dorsal pallium (dark blue: mesocortex; light blue: isocortex); LP: lateral pallium (green); MP: medial pallium (red); S: septum (yellow); St: striatum (purple); T: thalamus

The rostral expansion of the isocortex forms the frontal lobe in primates. It also expands posteriorly and caudally, incorporating the thalamus and flanking the brainstem. This expansion, along with that of the internal capsule and thalamus, deforms the deeper medial structures of the brain following a ring-like transformation. This causes a developmental rotation of the hippocampus, stria terminalis, tail of the caudate nucleus, temporal horn of the lateral ventricle, choroid plexus, corpus callosum, and cingulate gyrus, establishing what is known as the “limbic ring” (Figures 2 and 3) [18].

**Figure 4:**
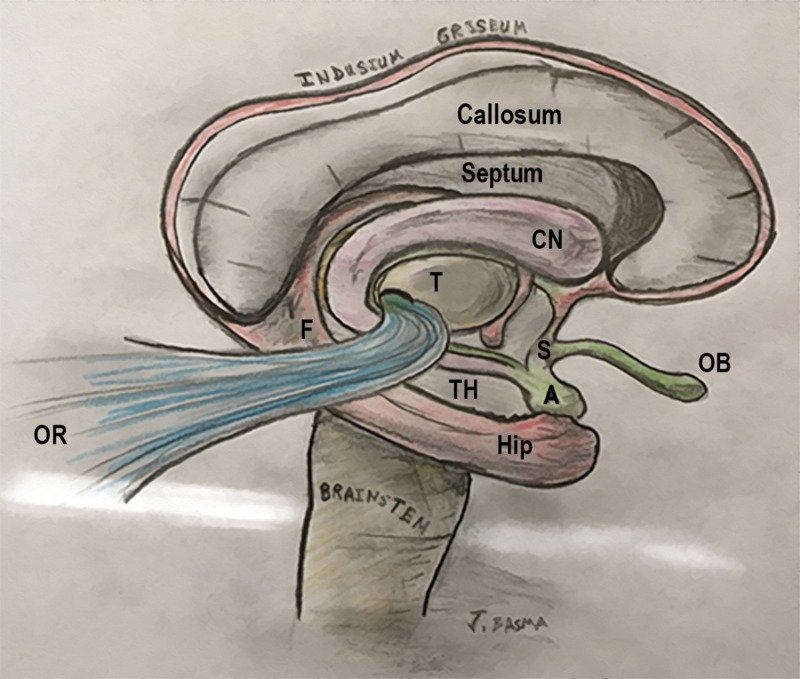
Illustration of the C-shaped structures of the limbic ring around the thalamus Benno Dukhor, professor of psychiatry at Basel, described the basic architecture of the human brain as multiple ram horns – i.e., the limbic and paralimbic structures – surrounding a smaller ram’s head – i.e., the stalk of the brain formed by the brainstem and thalamus. These “horns of the brain” include the fornix and the hippocampal formation, stria terminalis and amygdala, caudate nucleus and tail, choroid fissure, lateral ventricle, cingulate and parahippocampal gyri, corpus callosum, and the isocortical mantle (frontal, parietal and temporal lobes). They form incomplete rings, or C shapes, and their anterior non-curved parts span between the anterior perforated substance, uncus, and septal area. The C structures engulf the internal capsule from medial to lateral. While they are medial to the corticospinal tract rostrally, the rotation places them lateral to it inferiorly. For instance, the columns of the fornix are medial to the internal capsule, but the hippocampal head becomes lateral to the cerebral peduncles on the floor of the temporal horn A: amygdala; CN: caudate nucleus; F: fornix; Hip: hippocampus; OB: olfactory bulb; OR: optic radiations; S: septum; T: thalamus; TH: temporal horn

**Figure 5:**
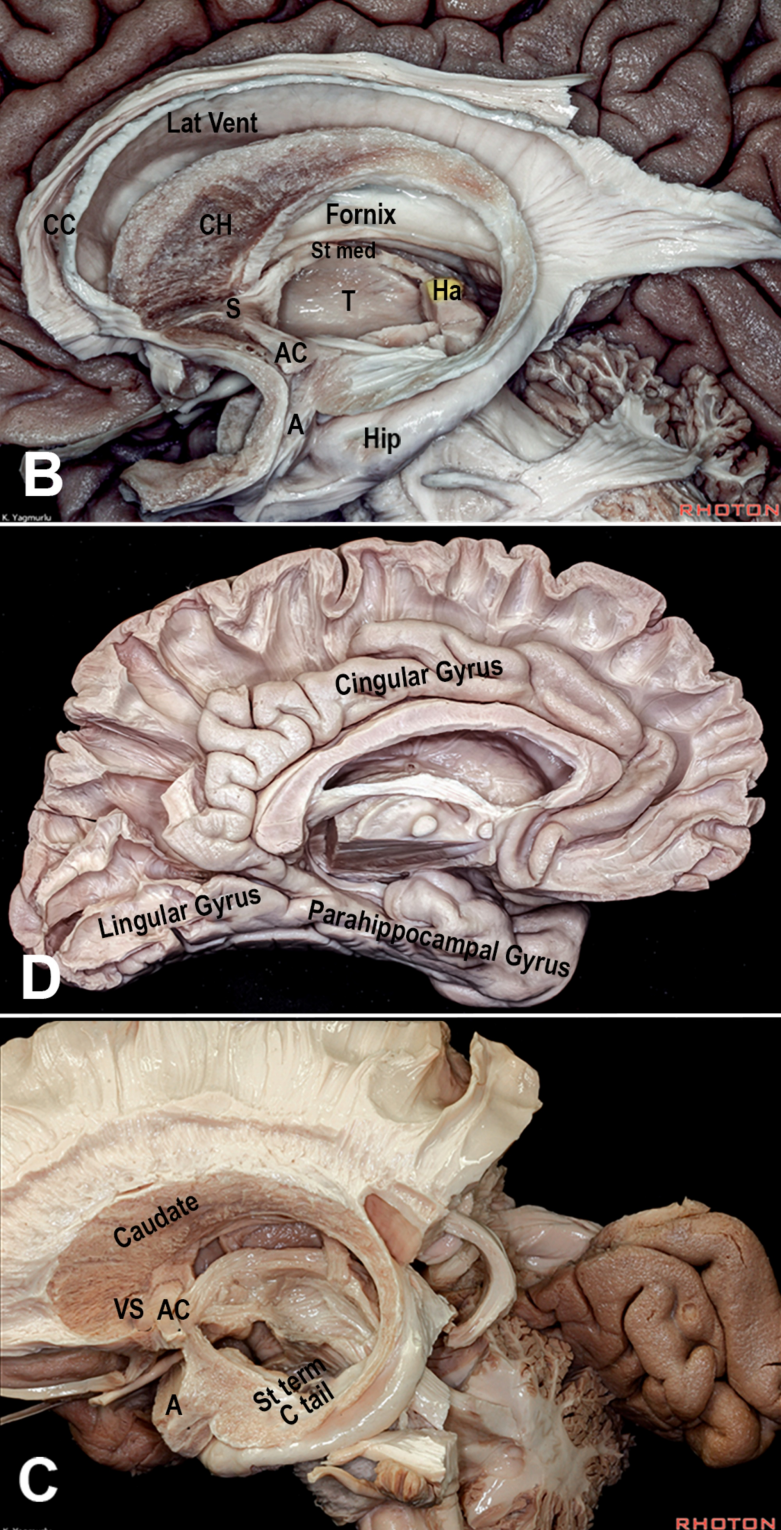
Dissections from the Rhoton Collection of the limbic system Also note the C-shape of the medial mesocortex (parahippocampal and cingulate gyri) A: amygdala; AC: anterior commissure; CC: corpus callosum; CH: caudate head; Ha: habenula; Hip: hippocampus; S: septum; St med: stria medullaris; T: thalamus Dissections by Kaan Yağmurlu. Courtesy of the Rhoton Collection

**Figure 6:**
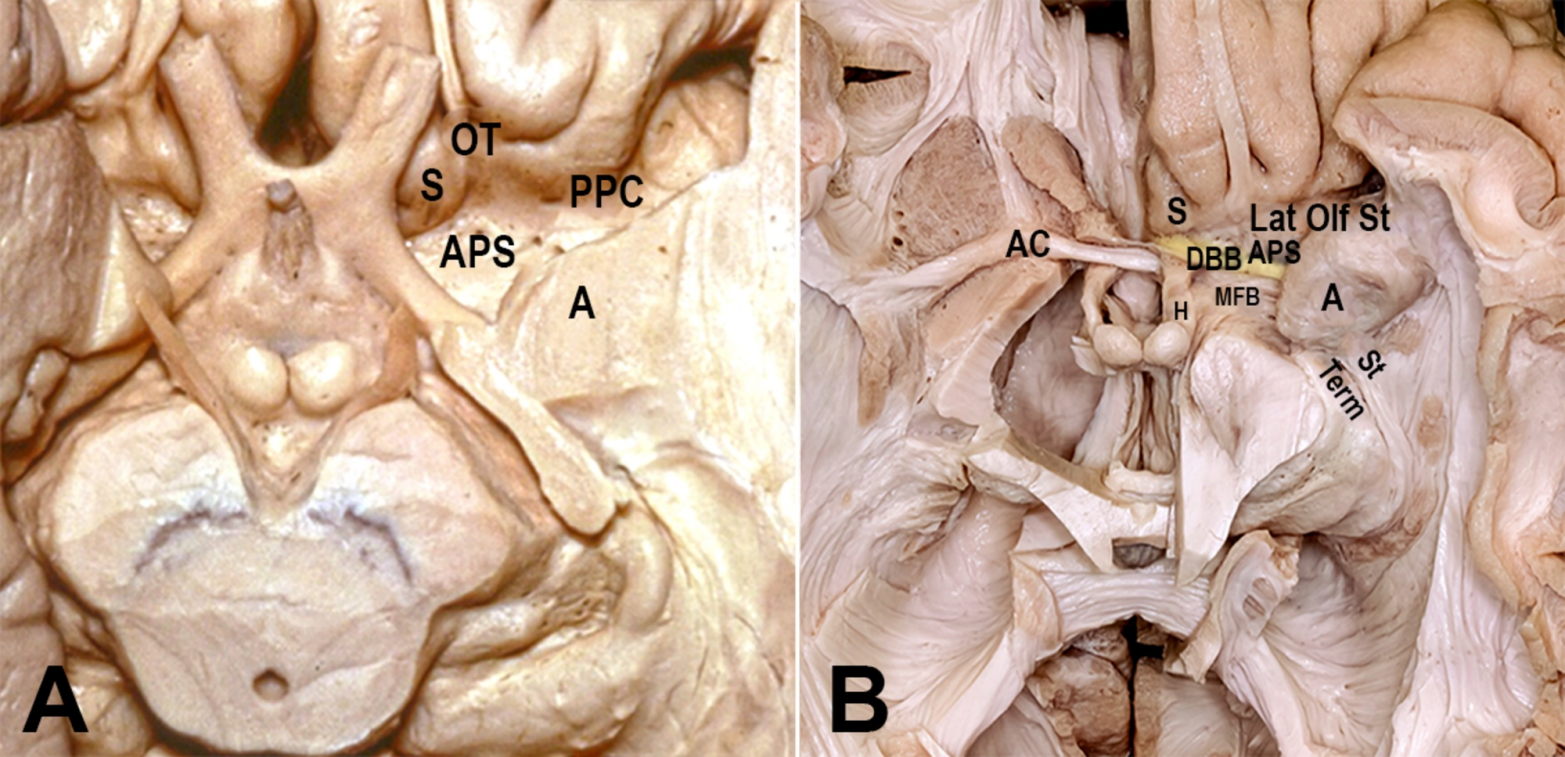
Dissections showing the fronto-basal region (A, B) In the area of APS, the lateral olfactory stria (Lat Olf St) courses lateral towards the olfactory cortex (peripiriform cortex, PPC), while the medial olfactory stria heads to the septum (S) and hippocampal formation. Between them are found the amygdaloid body and the septal area. Topologically, this region corresponds to the bordering zone between the medial pallium (hippocampus) and lateral pallium (olfactory cortex), which remained fixed anteriorly and were not influenced by the expansion of the dorsal pallium (isocortex). The two poles of the cerebral rotation can be deduced. At the rostral pole of the hemisphere, the piriform cortex (lateral pallium) and taenia tecta (precommissural hippocampus, medial pallium) converge. At the caudal pole, the posterior limit of amygdala (periamygdaloid cortex, lateral pallium) and the hippocampal head (medial pallium) are continuous in the uncus region A: amygdala; AC: anterior commissure; APS: anterior perforated substance; DBB: diagonal band of Brocca; H: hypothalamus; MFB: medial forebrain bundle; OT: olfactory tract; PPC: peripiriform cortex; S: septum; St Term: stria terminalis Dissections by Albert Rhoton Jr and Kaan Yağmurlu. Courtesy of the Rhoton Collection

Topological analysis of this rotation concludes that its fixed transverse axis passes through the region of the amygdala and limen insulae. It is believed that the rostral and caudal poles of the hemisphere were not deformed and remained static relative to each other [18]. At the rostral pole, the medial pallium and lateral pallium come into contact at the base of the septal area, between the peripiriform cortex (olfactory) and the precommissural hippocampus (Figures 4,5,6). On the other hand, the primordial caudal pole of the hemisphere is located where the periamygdaloid cortex (olfactory) and hippocampal head are in proximity (uncus). Hence, the regions of the uncus and the anterior perforated substance form the two ends of the “C” ring [4].

Topological Transformations of the Hippocampus

**Figure 7:**
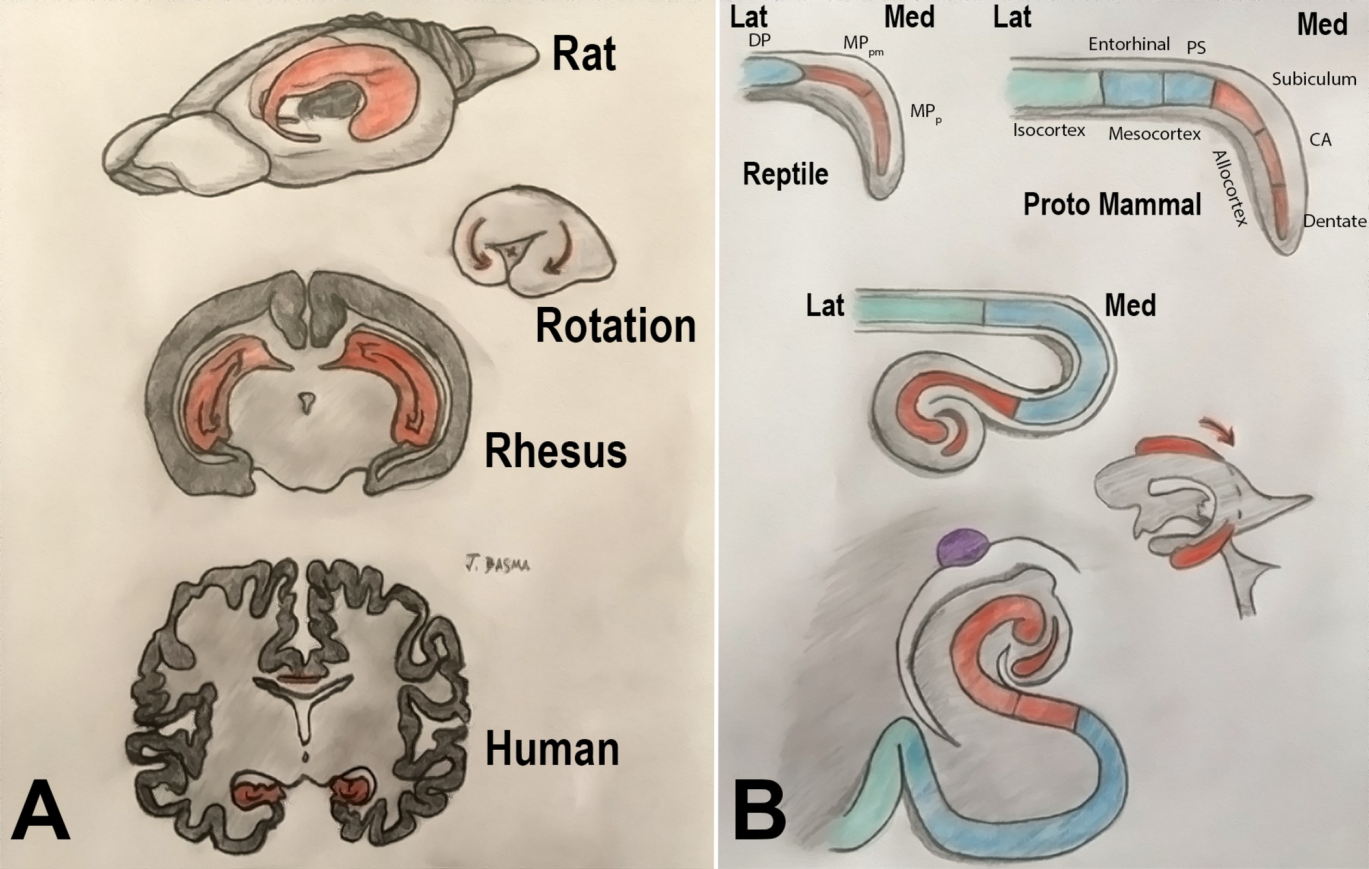
Development of the human hippocampus (A) Drawing illustrating the difference of hippocampal position between the rat, rhesus monkey, and human brains. (B) Folding of the hippocampus. The primitive medial pallium (MP) differentiates into two sectors: magnocellular (MPm) or hippocampus proper (cornu ammonis), and a parvocellular sector (MPp), precursor of the dentate gyrus. Since the dentate gyrus curls medially at the tip of the hippocampal formation, and the entorhinal cortex is also shifted medially, the cornu ammonis itself is moved laterally, forming an “S” shape on the coronal sections. The dentate gyrus loses its connection with the hippocampus proper and curls as an independent C structure around the end of the latter. The entorhinal cortex and the parahippocampal gyrus (from the dorsal pallium) become topographically medial relative to the hippocampus. The limbic rotation moves the hippocampus from the roof to the floor of the ventricle. (Adapted from Gloor) [4] CA: cornu ammonis; DP: dorsal pallium; Fimb: fimbria; Lat: lateral; Med: medial; MPm: medial pallium/magnocellular sector; MPp: medial pallium/parvocellular sector; PS: parasubiculum

**Figure 8:**
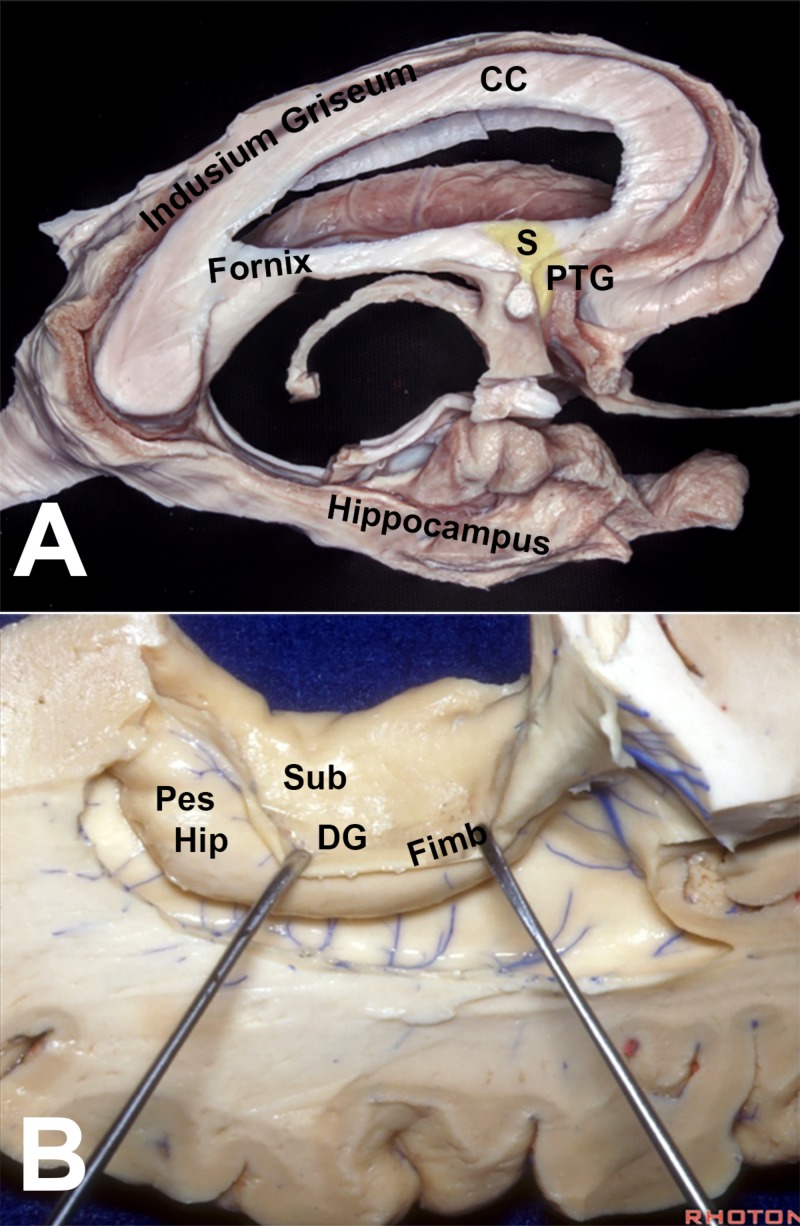
Dissections showing the different parts of the hippocampal formation (A,B) The subcallosal hippocampus is represented by the fornix, which encircles the thalamus before dividing into precommissural fibers (anterior to the anterior commissure, reaching the hippocampal tenia tecta and the septal area), and postcommissural fibers (destined to the mammillary bodies) CC: corpus callosum; DG: dentate gyrus; Fimb: fimbria; Pes Hip: pes hippocampi; PTG: paraterminal gyrus; S: septal area; Sub: subiculum Dissections by Albert Rhoton Jr and Kaan Yağmurlu. Courtesy of the Rhoton Collection

The mammalian hippocampus is distinguished by its twinned “interlocking C” morphology formed by the cornu ammonis and the dentate gyrus. The primitive medial pallium differentiates into two sectors: magnocellular or hippocampus proper (cornu ammonis), and a parvocellular sector, precursor of the dentate gyrus. Lateral to these structures, the medial pallium also contributes to the subiculum. The growing hippocampal formation-limited rostrally and laterally by the rest of the cerebrum folds around itself and establishes the hippocampal sulcus (between the dentate gyrus and the subiculum). The downward expansion of the isocortex causes the hippocampal formation to rotate downwards around the thalamus. Hence in non-primate mammals, the hippocampus occupies a rostral position in the brain. Being initially at the superior aspect of the hippocampus, the subiculum changes its topographic position with the hippocampal rotation to become ventral to the dentate gyrus, thus earning its Latin name, meaning “support” (Figures 7 and 8) [4,19].

Development of the Corpus Callosum

Like reptiles, acallosal mammals have their major commissural fibers crossing in the anterior commissure. The corpus callosum becomes the major bi-hemispheric relay after its fibers cross through the hippocampus. It has been postulated that the hippocampal commissure and the corpus callosum may have a common ancestral “pallial commissure” [20]. A hippocampal remnant, called the indusium griseum, is left on top of the corpus callosum and connects with the rest of the hippocampus through the gyrus fasciolaris. The subcallosal hippocampus is represented by the fornix, which encircles the thalamus before dividing into precommissural fibers (anterior to the anterior commissure, reaching the hippocampal tenia tecta and the septal area), and postcommissural fibers (destined to the mammillary bodies) (Figure 4C) [19]. The hippocampal layer between the corpus callosum and the fornix is elongated on both sides to form the septum pellucidum. When these bilateral layers are not fused, they form the cavum septi [20].

Choroid Fissure

The interventricular foramen, from which the telencephalic vesicle evaginated, remains close to its origin near the medial wall of the hemisphere. This wall is formed by the hippocampus and stria terminalis (medial pallium). Between these two structures, a thin layer of tissue does not develop into a functional element, but instead comes to rest on the thalamus [4]. It eventually folds to form the epithelialized choroid plexus. The lamina choroidea fuses with the thalamus, becoming the lamina affixa, which makes the thalamus (diencephalic) part of the floor of the lateral ventricle (telencephalic). Thus, the choroid plexus seems to append to the thalamus (taenia thalami), rather than the stria terminalis, and fornix (taenia fornicis). In the temporal horn, however, the choroid plexus keeps its original attachment to the stria terminalis [21].

**Figure 9:**
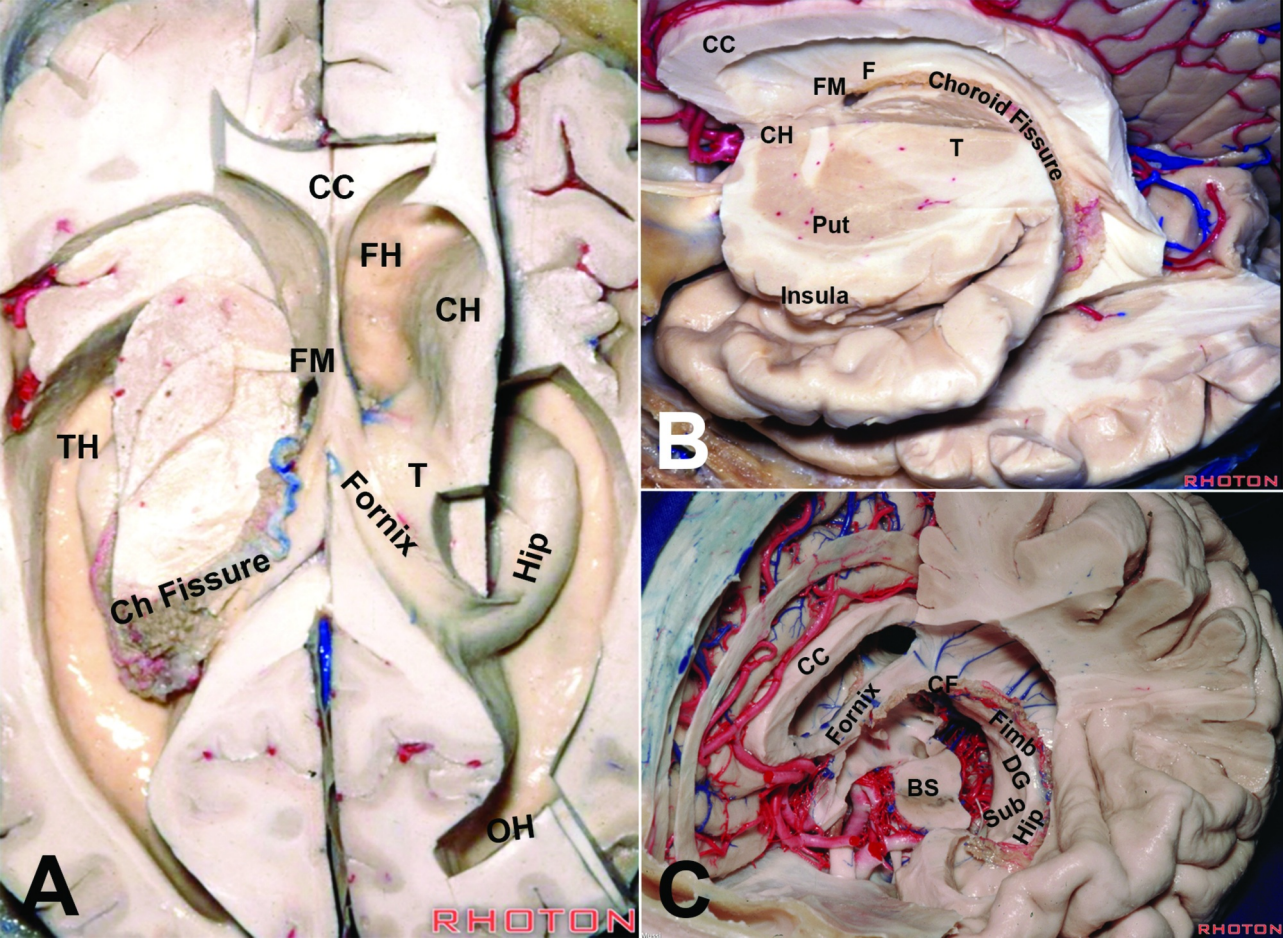
Dissections illustrating the topographic relationships of the ventricular walls and the choroid fissure (A–C) The choroid fissure extends between the fornix and the thalamus as a topological elongation of the foramen of Monro in the medial wall of the hemisphere BS: brainstem; CC: corpus callosum; Ch Fissure/CF: choroid fissure; CH: caudate head; DG: dentate gyrus; FH: frontal horn of the lateral ventricle; FM: foramen of Monro; Hip: hippocampus; OH: occipital horn; Put: putamen; Sub: subiculum; TH: temporal horn Dissections by Albert Rhoton Jr. Courtesy of the Rhoton Collection

The caudal rotation of the brain causes the ventricle to loop backwards and downward around the thalamus. Consequently, the choroid fissure gains its famous C-shape between the thalamus and the fornix as the medial wall of the lateral ventricle, and as a topological extension of the interventricular foramen (Figure 9) [4].

Gyrification of the Primate Cortex

Similar to its embryologic development, the olfactory cortex expands early and forms the piriform lobe. Dorsal to it, the isocortex develops and becomes separated from it by the rhinal sulcus. This sulcus is phylogenetically “old” and is the only apparent sulcus on the lateral convexity of lissencephalic mammals [22,23].

The isocortex then follows a large expansion relative to the allocortex, and folds around the rest of the brain. In primates, its downward growth leads to the formation of the temporal lobe, displacing the rhinal sulcus (Figure 6A). It is postulated that the evolution of vision and object recognition instigated the appearance of the associative cortex, including the temporal lobe [23]. Conceivably, this would explain why there is no constant sulcal separation between the temporal and occipital lobes.

**Figure 10:**
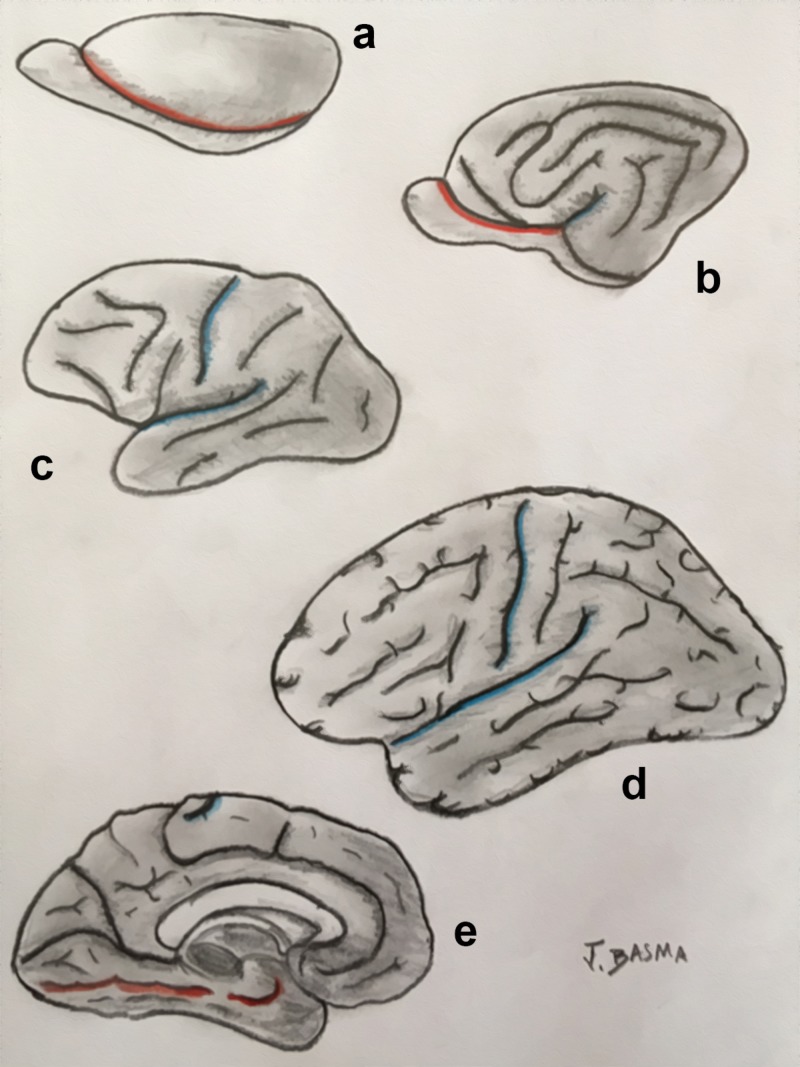
Illustration of the sulci and gyri of a rat, cat, monkey, and human Illustration of the sulci and gyri of a rat (A), cat (B), monkey (C), and human (D: lateral view; E: medial view). The rhinal sulcus (red) separates the olfactory cortex from the isocortex and is the only apparent sulcus on the lissencephalic rat brain. In humans it becomes continuous with the collateral sulcus, delineating the fusiform from the parahippocampal gyrus. The Sylvian fissure and the central sulcus are all hallmarks of primate brains

**Figure 11:**
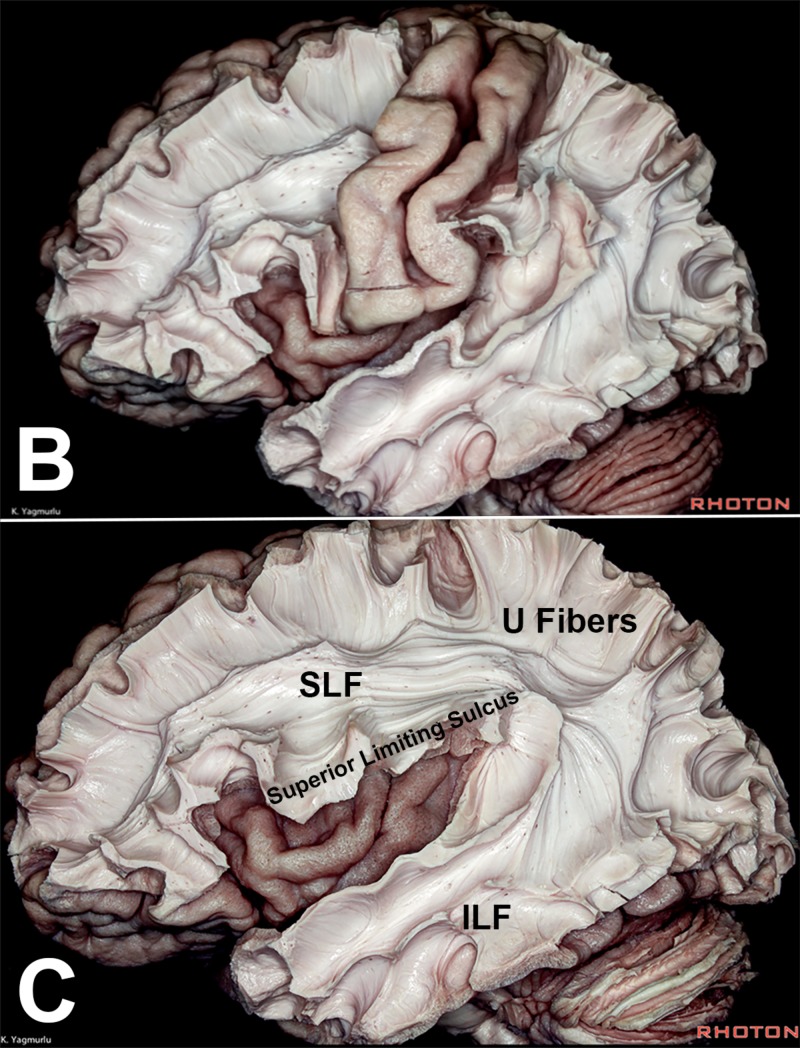
Dissections showing the primary motor and sensory regions around the central sulcus, and subcortical deep white matter fibers (A) The primary motor and sensory regions around the central sulcus, and (B) subcortical deep white matter fibers. Note the C-shape orientation of the ILF and SLF ILF: inferior longitudinal fasciculus; SLF: superior longitudinal fasciculus Dissections by Kaan Yağmurlu. Courtesy of the Rhoton Collection

The anterior expansion of the isocortex and thus of the frontal lobe occurred after the temporal lobe, and hence is more prominent in humans compared with other primates. The folding of the frontal and temporal lobes over the insula formed the opercula around the Sylvian fissure. Evolution of manual dexterity in primates was reflected by the development of the central lobe with its highly specialized somatosensory and somatomotor areas (Figures 10 and 11) [23,24]. Further expansion of the cortex in humans has caused its surface to form sulci and gyri. This might have economized and increased the efficacy of fiber connectivity.

Development of White Matter Tracts

The basic design of vertebrate white matter tracts includes projection fibers (olfactory stria, medial forebrain bundle and lateral forebrain bundle, i.e. internal capsule and optic radiations), association fibers (limbic and paralimbic tracts, superior and inferior longitudinal and uncinate fasciculi) and commissural fibers (anterior commissure and corpus callosum) (Figure 6C) [25]. With primate evolution, the relative white matter volume increased exponentially, reflecting the increased interconnectivity of the cortex and cognitive processing abilities of anthropoid primates.

Preservation of topology in white matter is more difficult to follow as fiber tracts can pierce through gray matter structures (e.g., internal capsule through striatum), and the number and pattern of interconnections and association fibers approach randomness (i.e., “dispersion”) [26]. However, several mechanisms have been shown to influence the relationship between the topology of gray matter and that of white matter, explaining the “targeted wiring” and predictable orientation of the tracts (radial glial cells, pioneer neurons, and scaling) [26,27]

Authors’ synthesis

Topology challenges us to understand the complex deformations of the human brain in a continuous space. Integrating the temporal dimension in the anatomical description simplifies difficult three-dimensional topographic relationships. A “four-dimensional” anatomical presentation offers a dynamic model in which the static cerebral structures can be seen in their developmental movements.

**Figure 12:**
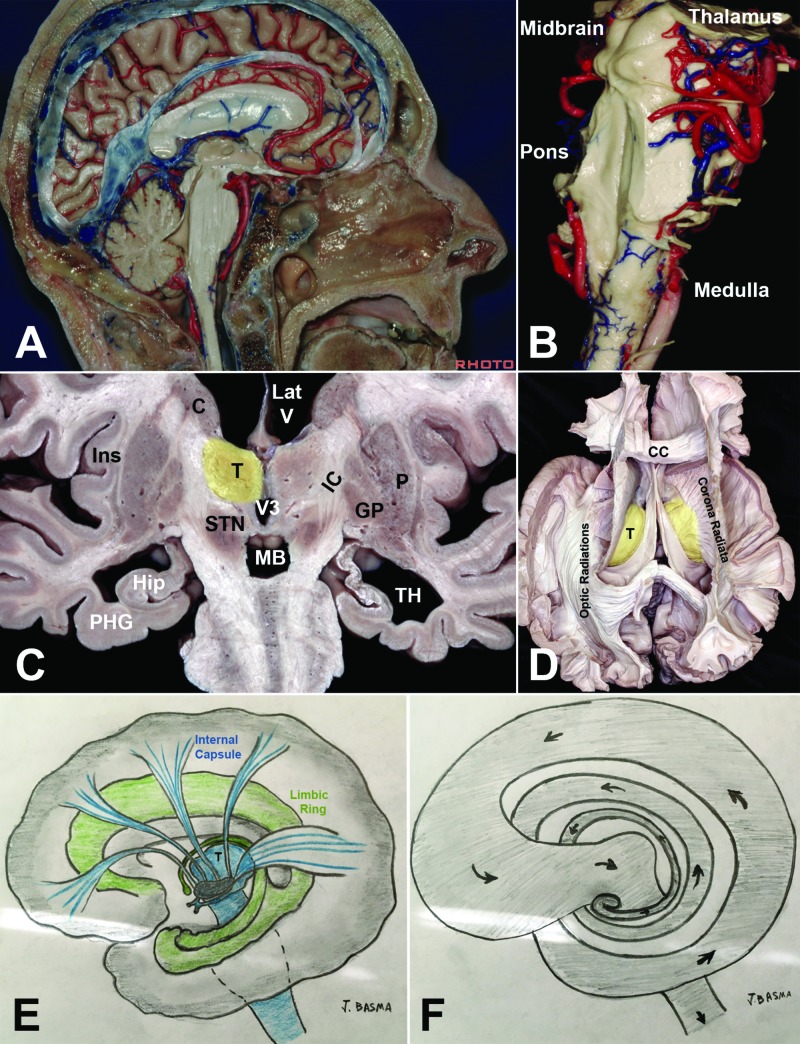
Synthesis of basic brain architecture The thalamus (T) is continuous with the brainstem (A, B) forming the stalk of the brain. The cortical fibers of the internal capsule (IC) connect with the thalamus (thalamic radiations) or drape it laterally to reach the cerebral peduncles (C). The corona radiata and the optic radiations establish immediate connections between the stalk of the brain with the superficially located cortex (D); the latter having migrated from the deep ventricular surface making room for subcortical white fiber tracts. (D) As the internal capsule and the thalamus enlarge with the cortical expansion, the structures beneath which they course are stretched posteriorly and caudally, forming the limbic and paralimbic rings. The limbic ring rotates from medial to lateral around the cortical fibers (internal capsule and optic radiations). (F) Continuity is not broken between the different circuits of the limbic ring and with the different isocortical layers. In a two-dimensional projection, this can be assimilated to a continuous topological “cerebral strip.” C: caudate; CC: corpus callosum; GP: globus pallidus; Hipp: hippocampus; IC: internal capsule; Ins: insula; Lat V: lateral ventricle; P: putamen; PHG: parahippocampal gyrus; STN: subthalamic nucleus; T: thalamus; TH: temporal horn of the lateral ventricle Dissections by Albert Rhoton Jr, Antonio Mussi, and Kaan Yagmurlu. Courtesy of the Rhoton Collection

The regions of medial, lateral, and dorsal pallia should be conceptualized in a three-dimensional elliptical telencephalic vesicle in which the medial and lateral pallia meet rostrally and caudally. Through time, the dorsal pallium sprouts out dorsally, laterally, and posteriorly relative to the neighboring regions, while maintaining a direct connection with the stalk medially (via the internal capsule) (Figure 12). Like an umbrella, the cortex comes to drape over the entire structure, while its stalk (internal capsule) stretched the limbic ring from within. In this process of cortical migration, the superficially located cortex gave room for white matter to increase the density of remote brain interconnections. Optic radiations (which are part of the retro-lenticular internal capsule emerging from the lateral geniculate body), loop anteriorly before coursing posteriorly towards the occipital cortex (Meyer’s loop). They curve around the limbic ring, instead of penetrating it and interrupting its continuity.

The lateral pallium, developing less significantly than the other pallia, is forced into the basal part of the brain. With the regression of the olfactory vesicle, it eventually loses its periventricular position.

The corpus striatum is on the floor of the telencephalic vesicle. It is pushed further in and down by the expanding cortex and almost fuses with the thalamus. The part of the striatum dorsal to the capsule, the caudate, undergoes a “ram horn rotation.” The ventral part (the lenticular nucleus) is not affected by the rotation. The caudate and the lenticular nucleus meet anterior to the internal capsule, at the region of nucleus accumbens. While the thalamus becomes incorporated into the floor of the ventricle medially, the growing head and body of the caudate come to form the lateral wall of the ventricle. The choroid fissure expands almost as an elongation of the foramen of Monro.

The fast-growing frontal and temporal lobes form opercula on either side of the Sylvian fissure while remaining continuous through the slower-growing insula [23]. This continuity is also remarkably preserved in the C-shaped structures of the limbic system. The areas where the lateral and medial pallia meet form the primordial rostral and caudal poles of the hemisphere around which the C-shape rotations occur.

The mesocortex, or paralimbic cortex, is a transitional area between the six-layered granulated isocortex and the three-layered allocortex. Medially, it includes the parahippocampal gyrus, the entorhinal cortex, and the cingulate gyrus. Laterally, it includes the insula and orbitofrontal cortex. Continuity is established between the medial and lateral mesocortical at the surface of the uncus (peripiriform cortex and parahippocampus) and at the subcallosal-mesial orbitofrontal region (between cingulate gyrus and orbitofrontal gyrus). Multiple areas of transition from allocortex to mesocortex are seen (e.g., between the hippocampus and entorhinal cortex, and between the orbitofrontal cortex and piriform cortex/septal area). The mesocortex also transitions to the isocortex in multiple regions (e.g., cingulate gyrus and precuneus, orbitofrontal cortex and frontal gyri, parahippocampal gyrus and fusiform/temporal gyri, etc).

Thus, the C-shape structures should not be conceived as dead-end extensions from their precursors. The primitive structures are grown and deformed, yet they maintain their original neighboring relationships. Continuity is not preserved through a “cut and paste” process, but through “deformational scaling.” When we examine the “ram horn rotations” of the brain and project them to a two-dimensional sagittal plane, we obtain a “cerebral strip,” where the brain seems to rotate around its stalk, from medial to lateral, along a continuum of different functional systems (Figure 12).

## Discussion

Repercussions of phylogenetics in neurological and neurosurgical pathologies have been observed. For instance, Friedrich’s ataxia affects the mammalian dorsal spinocerebellar tracts, dorsal columns, corticospinal tracts, and neocerebellum. Pick’s disease is seen in primate-specific structures (i.e., the frontal and temporal lobes), and spares the primary sensory and motor cortices. Krabbe’s leukodystrophy involves subcortical white matter (of the isocortex) but spares allocortical fibers, such as the olfactory tracts, fornix, and mammillothalamic tract [3].

Through millennia of cerebral topological development, anatomical lines of separation between the brain’s structures have been established. These lines retrace the historical trails of phylogeny. With the agility of the modern neurosurgeon, they have become surgical planes that can be safely unlocked as corridors to deep cerebral structures. For instance, the Sylvian fissure is a workhorse for neurosurgical approaches to the circle of Willis and the upper brainstem [2]. The choroid fissure can be opened to access the third ventricle, pineal region, and peri-mesencephalic cisterns. Trans-sulcal approaches have been employed to spare parenchymal disruption [28]. While medial sulci follow the rotation of the limbic rings and are oriented in a C-shape configuration (e.g., cingulate sulcus), lateral sulci, on the other hand, were formed by the process of cortical gyrification, and are oriented towards the ventricular cavity from which primordial cortical cells originally migrated. Benign and early malignant neocerebral glial tumors grow following the basic principles of topology and deform the normal topographic anatomy of the brain [2]. Understanding pathological topology is crucial for planning the surgical approach.

Since both evolutionary and neoplastic processes involve “mutations,” it is theorized that tumors may be accidental side effects of evolution. Brain tumors appear to have a predilection for specific regions of the brain [29]. Yasargil argued that such preferential cartography is related to distinct cytoarchitectonic properties and phylogenetic origins [2]. Brodman discovered that architectonic differences in particular cortical regions correlate with their functional organization [24]. This cortical variation is hypothesized to be associated with regional differences in glial architecture and biology. Glial cells play a role in neuronal migration, regulation of synaptic transmission, and metabolism. Yasargil observed that glial tumors tend to occur in phylogenetically “recent” systems (i.e., the association cortex) or “older constantly active” regions (i.e., those involved in memory, or limbic system) [2].

Diffuse low-grade gliomas involving the supplementary motor area (SMA) and insula were noticed to be infiltrative, spreading along white matter fibers (uncinate fasciculus and superior longitudinal fasciculus). Both the insula and the SMA share transitional cytoarchitectonic and functional characteristics between an agranular primary unimodal cortex and a granular heteromodal association cortex, which may predispose them to a neoplastic process [30].

We could speculate that low-grade glial tumors, in general, may have a predilection for cytoarchitectonic and functional regions of transition. This holds true even when we consider Yasargil’s classification in light of the topological development of the brain [2]. The temporal mediobasal region (limbic type 1) harbors a continuation between the allocortex (hippocampus and amygdala) and the mesocortex of (parahippocampal and cingular gyri). The cingulate gyrus (type 2) has transitional areas with the precuneus and different frontoparietal isocortical regions. The mesocortical insular cortex (type 3) is continuous with the frontotemporal opercula and the fronto-orbital cortex. At the fronto-basal region (type 4), the fronto-orbital mesocortex meets the frontal isocortex and the olfactory and septal allocortices. While aneurysms commonly develop at sites of arterial branching (Rhoton), glial tumors seem to have a propensity for areas where there is a shift of function or histology along the cerebral continuum. Such speculation needs to be tested against present and future genetic and molecular research.

## Conclusions

Drawing from classical neuroanatomical literature and modern theories describing the complex topological transformations of the brain, phylogenetics, comparative neuroanatomy, and updated theories of white matter development, cerebral anatomy can be studied through the fourth dimension of time, and a speculated element of causality.
